# The Effects of Single Instance Transcutaneous Vagus Nerve Stimulation on Cognition in MCI: A Randomized, Double‐Blind, Placebo‐Controlled Crossover Trial

**DOI:** 10.1002/alz.090449

**Published:** 2025-01-09

**Authors:** Brian D. Ho, Xavier A. Velez, Abigail B. Waters, Alexandria G. O'Neal, Damon G. Lamb, Steven T. DeKosky, Eric S. Porges, Ronald A. Cohen, John B. Williamson

**Affiliations:** ^1^ University of Florida, Gainesville, FL USA

## Abstract

**Background:**

Transcutaneous stimulation of the auricular branch of the vagus nerve (tVNS) was administered to participants diagnosed with mild cognitive impairment (MCI) to improve word‐list memory (primary outcome) and other cognitive skills.

**Method:**

A randomized, double‐blind, placebo‐controlled crossover design was used for this trial. Participants with MCI (n = 59) were sorted into one of two sequences: Sham‐tVNS or tVNS‐Sham. In the Sham condition, electrodes were placed on opposite sides of participants’ left earlobes; the return electrode was placed on the mesial face of the earlobe. In the tVNS condition, electrodes were placed over the left auricular branch of the vagus nerve with the return electrode placed anterior to the tragus and one placed on the posterior face of the tragus to the interface of the auditory canal. In both conditions, stimulation parameters were set to 20Hz, 100 µs pulse width of alternating polarity, and intensity ramped from 0 to the threshold of discomfort and then reduced by 20%. During stimulation, participants simultaneously underwent comprehensive neuropsychological testing to assess various cognitive abilities. Test batteries were identical at both study visits, which were separated by at least 72 hours to avoid carryover effects, except where alternative forms were available. Structural brain MRI was also acquired.

**Result:**

Performance on the immediate and delayed recall trials of the Rey Auditory Verbal Learning Test (RAVLT) did not significantly differ by treatment condition (*p* = 0.072 and *p* = 0.279, respectively). Likewise, performance on other cognitive tests did not significantly differ between treatment conditions (*p* = 0.101 to 0.958).

**Conclusion:**

A single bout of tVNS does not appear effective for improving cognition in individuals with MCI.

